# Comparison of multistate Markov modeling with contemporary outcomes in a reanalysis of the NINDS tissue plasminogen activator for acute ischemic stroke treatment trial

**DOI:** 10.1371/journal.pone.0187050

**Published:** 2017-10-26

**Authors:** Christy Cassarly, Renee’ H. Martin, Marc Chimowitz, Edsel A. Peña, Viswanathan Ramakrishnan, Yuko Y. Palesch

**Affiliations:** 1 Department of Public Health Sciences, Medical University of South Carolina, Charleston, South Carolina, United States of America; 2 Department of Otolaryngology–Head & Neck Surgery, Medical University of South Carolina, Charleston, South Carolina, United States of America; 3 Department of Neurology, Medical University of South Carolina, Charleston, South Carolina, United States of America; 4 Department of Statistics, University of South Carolina, Columbia, South Carolina, United States of America; University of Glasgow, UNITED KINGDOM

## Abstract

Historically, ordinal measures of functional outcome have been dichotomized for the primary analysis in acute stroke therapy trials. A number of alternative methods to analyze the ordinal scales have been proposed, with an emphasis on maintaining the ordinal structure as much as possible. In addition, despite the availability of longitudinal outcome data in many trials, the primary analysis consists of a single endpoint. Inclusion of information about the course of disease progression allows for a more complete understanding of the treatment effect. Multistate Markov modeling, which allows for the full ordinal scale to be analyzed longitudinally, is compared with previously suggested analytic techniques for the ordinal modified Rankin Scale (dichotomous-logistic regression; continuous-linear regression; ordinal- shift analysis, proportional odds model, partial proportional odds model, adjacent categories logit model; sliding dichotomy; utility weights; repeated measures). In addition, a multistate Markov model utilizing an estimate of the unobservable baseline outcome derived from principal component analysis is compared Each of the methods is used to re-analyze the National Institute of Neurological Diseases and Stroke tissue plasminogen activator study which showed a consistently significant effect of tissue plasminogen activator using a global test of four dichotomized outcomes in the analysis of the primary outcome at 90 days post-stroke in the primary analysis. All methods detected a statistically significant treatment effect except the multistate Markov model without predicted baseline (p = 0.053). This provides support for the use of the estimated baseline in the multistate Markov model since the treatment effect is able to be detected with its inclusion. Multistate Markov modeling allows for a more refined examination of treatment effect and describes the movement between modified Rankin Scale states over time which may provide more clinical insight into the treatment effect. Multistate Markov models are feasible and desirable in describing treatment effect in acute stroke therapy trials.

## Introduction

The modified Rankin Scale (mRS) is the most commonly chosen primary outcome measure in clinical trials of acute stroke therapy [1]. Despite the ordinality of the outcome measure, many trials have dichotomized the mRS for the primary analysis [2, 3]. In general, ignoring these differences and dichotomizing does not allow for examination of the treatment effect at finer gradients of the scale [4]. Any reduction in power may result in failure to find a clinically meaningful treatment effect during analysis of the data.

A number of alternative methods have been proposed to improve the analysis of the mRS. Some trials have analyzed the mRS as a continuous outcome, utilizing t-tests or linear regression [5]. Other trials have used the Cochran-Mantel Haenszel (CMH) shift test to analyze the distribution of the mRS, where the primary outcome is a favorable shift toward better functional outcome [2]. Ordinal logistic regression has also been proposed and demonstrated in re-analysis of stroke trial data [6]. The proportional odds model (POM) has been used but the test for the proportional odds assumption is not well-powered. In cases where the assumption was not justifiable, the partial proportion odds model (PPOM) or the adjacent categories logit (ACAT) has been used [6]. A popular alternative to continuous, ordinal and strict dichotomous analysis is responder analysis or the sliding dichotomy, where the definition of success is allowed to vary depending on baseline severity [7]. Most recently, a utility weighted mRS (UW-mRS) was derived to provide a patient centered metric of the degree of benefit or harm of a treatment that can be analyzed with a t-test or linear regression [8].

A drawback of the outcome measures and analytic strategies listed above is that each analyzes data from a single endpoint, commonly the 90 day outcome, for the primary analysis despite the availability of repeated response measures collected over the course of longitudinal follow-up. A literature search for repeated measures analysis of acute stroke trial data returned only two articles where a generalized estimating equations approach was used for repeated measures analysis of the mRS [9, 10]. There has been some evidence that there is marginal improvement in motor recovery 30 days after stroke [11]. In spite of this finding, there is evidence that the small improvement beyond 30 days may still be clinically meaningful [12] and some argue that there is a compelling enough reason to extend assessment of outcome up to 12 months because there are subgroups of patients that have delayed recovery and it allows for demonstration of sustainability [13].

Most recently, the Multistate Markov model (MSMM) was proposed for analysis of the mRS [14]. The MSMM analyzes repeated measures data with ordinal outcomes. These types of models describe how a subject moves between a series of disease states over time, which is desirable in the description of disease processes that naturally move through increasing stages of severity [15]. Results suggest that the MSMM can be a more efficient approach than dichotomized methods to analyze the mRS data in some scenarios [14].

MSMMs can provide a better clinical understanding of the disease process since the information from the entire course of the disease is used to estimate the parameters of the model. Another benefit of MSMMs is the potential for decreased sample size. The power of MSMMs applied to acute onset clinical trial data was shown to increase significantly when the number of follow-up visits was increased [14]. Future trials could collect the mRS more frequently, increasing the power to detect differences using this modeling technique. This could be more cost-effective than recruiting more subjects to increase power.

The purpose of this article is to demonstrate the MSMM as an approach for analysis of the mRS. The MSMM and the alternative methods listed above will be used to re-analyze the National Institute of Neurological Disorders and Stroke (NINDS) tissue plasminogen activator (t-PA) trial data. The results from each of the analytical analysis approaches will be compared with the results using the MSMM approach.

## Materials and methods

### Trial data

The seminal NINDS t-PA trial showed a consistently significant effect of t-PA using a global test of four outcomes (Barthel Index, mRS, Glasgow Outcome Scale and NIHSS) in the analysis of the primary outcome at 90 days post-stroke [16]. In addition to the 90 day primary outcome assessment, the mRS was also collected at 7–10 days, 180 days and 360 days from randomization.

Acute stroke requires immediate attention and treatment, posing a challenge to assess baseline outcome measures for clinical trials. Thus, the mRS is not obtainable immediately after the stroke at baseline and most often analysis is adjusted for baseline severity using the NIHSS [17]. Much of the progression or recovery experienced by a patient suffering from an acute onset disease is expected to occur early on. Moreover, typically, the goal of a treatment or therapeutic action is improvement in patient health compared to their baseline measure. To accurately quantify improvement, a measure of the outcome at baseline is ideal.

A prediction procedure using principal component analysis (PCA) for data reduction of baseline variables known to be correlated with functional ability was used. Briefly, PCA is a statistical data reduction method that, when applied to a large set of variables, can group correlated variables into a smaller set of important independent composite variables, or components. The PCA grouped measures of severity (individual items of the NIHSS) and other baseline variables known to be associated with functional ability (age, baseline glucose, time from stroke onset to randomization and the Alberta Stroke Program Early CT score) into components to calculate component scores [18]. The resulting component scores were used to assign the latent baseline mRS score and thus creating a comparable baseline mRS for analysis purposes.

### Multistate Markov models

In this paper, continuous-time MSMMs are used to describe the progression and recovery between mRS levels, or the disease states, over time. The main assumption of the MSMM is that the probabilities governing the transition between states only depend on the current state occupied by an individual. For example, the probability of a patient transitioning at 180 days only depends on the state the patient was in at 90 days.

**Fig 1:**
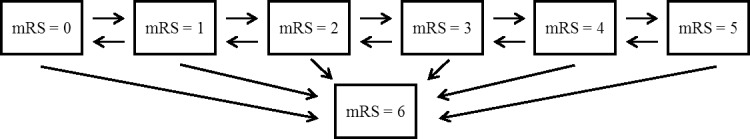
General MSMM for panel observed mRS data.

Death (mRS = 6) is known as an absorbing state because transitions out of this state cannot occur and mRS scores of 0 to 5 are examples of transient states, where transitions are allowed between the states. The data from the NINDS t-PA trial were observed at arbitrary times that were specified in advance so exact times of state transitions are unknown. Data of this type, observations of a continuous process at discrete times, are called panel data. Because the underlying disease process is continuous, where progression or recovery can happen at any time, it is assumed that in order for a subject to transition from one state to a non-adjacent state they also transition through the intermediate states [19]. Instantaneous transitions are only plausible between adjacent states because it is assumed that, for example, a subject that had an observed mRS score of 3 at one visit and an observed mRS score of 1 at the next visit must have also gone through 2 in the interval. Thus, the general MSMM for panel data only estimates adjacent state transitions and transitions to death from any state. The allowable transitions between transient and absorbing states for the general model of the mRS are illustrated in Fig 1, where arrows indicate the allowed transitions between states.

MSMMs of panel data are governed by transition intensities that depend on time and individual level or time-dependent covariates. The transition intensities represent the instantaneous risk of transition between two mRS scores. Commonly, the transition intensities are assumed to be constant over time but this is often an unrealistic assumption. If the assumption fails, a model with piecewise-constant transition intensities can be used. This allows for the transition intensity matrices to change at breakpoints, remaining constant between the breakpoints. In addition to transition intensities, transition probabilities can also be estimated based on the observed transition rates using maximum likelihood estimation [19]. When modeling covariates in a MSMM, hazard ratios can be estimated that correspond to the effect of a covariate on the transition intensities.

Likelihood ratio test (LRT) statistics are used to compare nested MSMMs. A reduced model is nested in a more complex model if all of the terms in the smaller model occur in the larger model. If two nested models are compared and the test is significant, then the more complex model fits the data better than the reduced model. LRTs are used to determine the significance of covariates and to compare models with constant transition intensities to ones with piecewise-constant intensities.

### Statistical analysis and assumptions

For analyses using a fixed dichotomy, favorable outcome was defined as mRS ≤ 1, as was done in the primary paper [20]. The PPOM includes an additional term using a second parameter that allows for the ORs to increase proportional to the outcome scale. This PPOM, the restricted PPOM, is used when there is a linear deviation from the proportional odds assumption required for the POM, which is true of the NINDS t-PA data [6]. For the sliding dichotomy analysis, favorable outcome was defined to be consistent with previous re-analysis of the NINDS t-PA data where mRS = 0 for subjects with mild stroke (NIHSS < 7), mRS ≤ 1 for subjects with moderate stroke (NIHSS = 8–14) and mRS ≤ 2 (NIHSS > 14) [21]. The UW-mRS values were derived by averaging patient centered and person-tradeoff studies and are reported by Chaisinanunkul et al [8].

The ACAT and MSMMs were fit in R statistical software version 3.4.1 using the *VGAM* and *msm* packages, respectively. All other analysis was completed in SAS 9.4. When appropriate, analyses were adjusted for baseline NIHSS, which is known to be highly predictive of outcome [22]. The model using responder analysis as well as the MSMM with predicted baseline mRS did not include baseline NIHSS because baseline severity is already accounted for. The shift analysis was also not adjusted for baseline severity as the test does not accommodate continuous covariates. Shift analysis for the NINDS t-PA data was previously repeated for different stratifications of the NIHSS and the results are reported elsewhere [2].

## Results

The analysis presented in this section is based on 619 subjects that had mRS scores recorded at 90 days. The raw 90 day mRS outcome distributions for the placebo and t-PA groups are presented in Table 1. There are slight differences in the results presented in this section compared with other re-analyses of the trial because the raw observed values are used rather than the intent to treat imputation.

**Table 1:** NINDS t-PA 90 day mRS counts (%).

Control	33 (5.3)	50 (8.1)	37 (6.0)	45 (7.3)	61 (9.9)	21 (3.4)	63 (10.2)	310
Treatment	57 (9.2)	74 (12.0)	23 (3.7)	40 (6.5)	42 (6.8)	19 (3.1)	54 (8.7)	309
Total	90	124	60	85	103	40	117	

**Fig 2:**
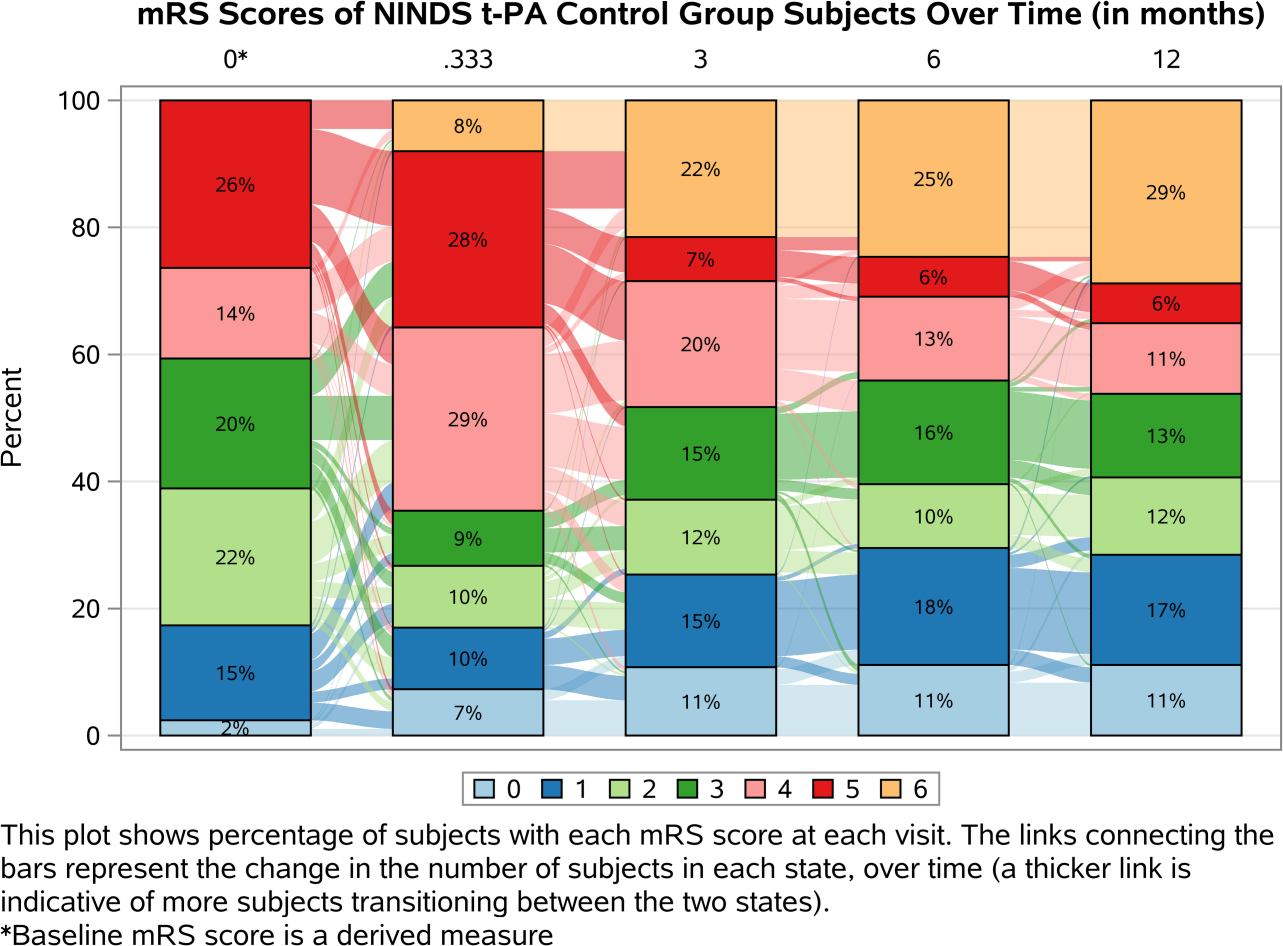
Sankey plot of NINDS t-PA control group mRS scores over time (months).

**Fig 3:**
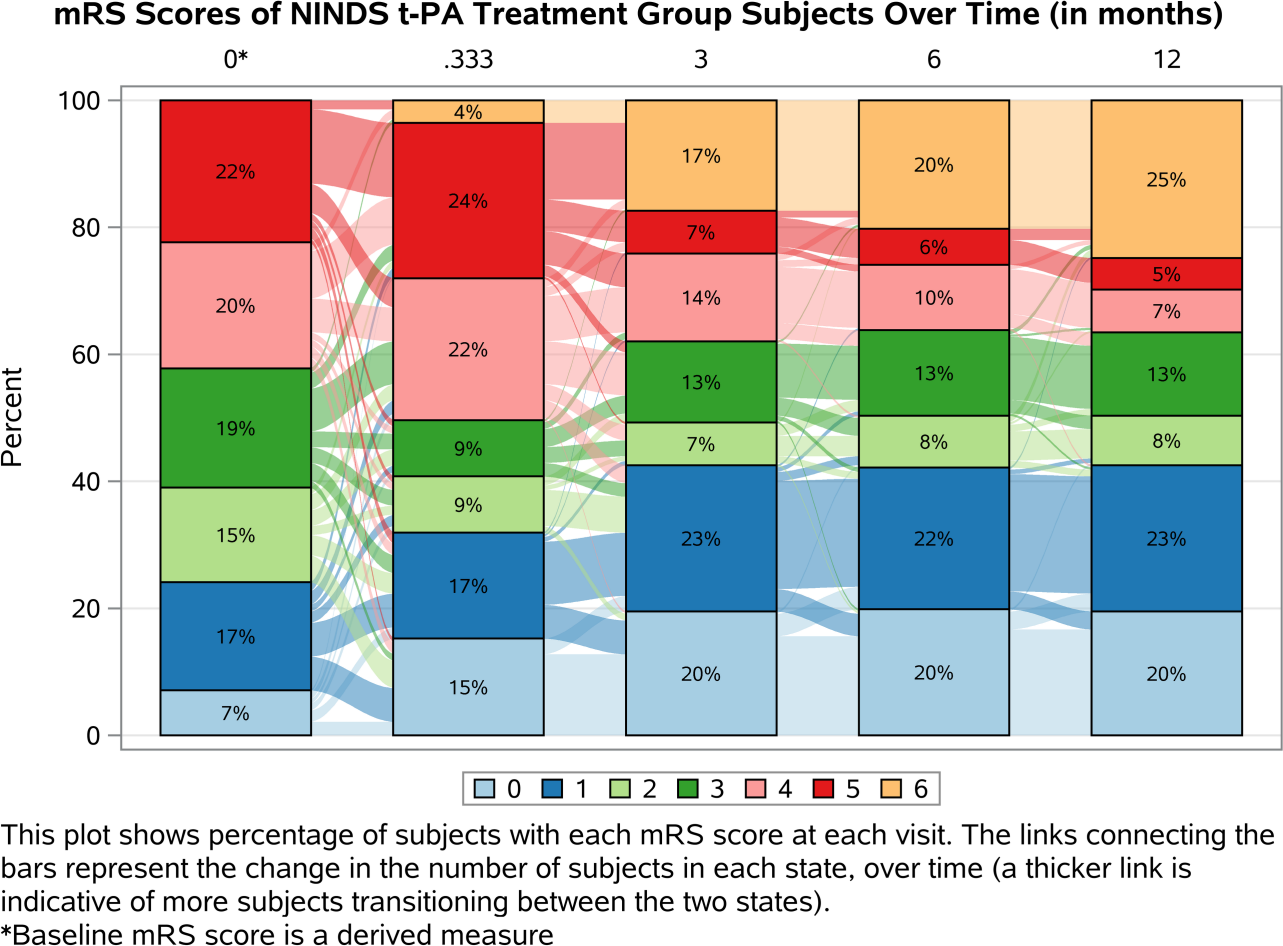
Sankey plot of NINDS t-PA treatment group mRS scores over time (months).

Figs 2 and 3 display mRS scores over time for the control and t-PA groups, respectively. These plots, called Sankey plots, show the percentage of subjects with each mRS score at each follow-up visit as well as the change in the number of subjects with each score over time [23]. In the 90 day mRS alone, there are differences between the groups across the entire ordinal scale that are ignored in a traditional dichotomized analysis. The use of one follow-up visit also results in a loss of information as there are differences in the distribution of the mRS as well as the transition rates over the entire follow-up period. For example, as displayed in the plots, the t-PA group has more favorable outcomes than the control group over time and we see that more deaths occur in the control group throughout the 12 month period. Additionally, the inclusion of the predicted baseline mRS allows one to observe the differences in the transition rates between treatment groups in the crucial window immediately following randomization and during the acute treatment phase. All of these differences can be measured and described using MSMMs and are not accounted for using other ordinal data analysis methods.

In the general MSMM (Fig 1), some of the parameters estimated were close to zero. Specifically, the transition intensities to death from mRS = {0, 1, 2, 3} were all very small. When there is not enough information from the data on certain transition rates, more intensities may need to be set to zero [19]. Thus, the general model was reduced, no longer allowing death from any state. Constraints were imposed such that death is only allowable from mRS = 4 or mRS = 5.

The results from all methods are presented in Table 2. The results are consistent with previously reported re-analyses of the NINDS t-PA data with minor, insignificant differences in estimates due to the adjustment for the NIHSS and the use of the raw mRS data versus intent to treat [6, 8, 9, 24, 25]. Table 3 presents a review of the interpretation of the summary statistics obtained from each of the methods of analysis. The results of the MSMM are presented as hazard ratios that estimate the effect of the covariate on transition intensities. A hazard ratio above one signifies a positive association between treatment and the rate of transition, whereas a hazard ratio of one implies no effect.

**Table 2:** Results from previously used methods for analysis of the mRS.

Method	Outcome Measure	Summary Statistic	(95% CI)	P
Logistic regression	mRS at 90 d (0–1 vs. 2–6)	OR = 2.04	(1.39, 2.99)	0.0003
Linear regression	mRS at 90 d (continuous)	Diff. in means = 0.50	-	0.0073
Shift analysis	mRS at 90 d	-	-	0.0017
POM	mRS at 90 d	OR = 1.41	(1.01, 1.81)	0.0172
PPOM (linear trend)	mRS at 90 d	OR =		0.0017
	0 vs. 1–6	1.88	(1.14, 2.61)	
	0–1 vs. 2–6	1.67	(1.12, 2.21)	
	0–2 vs. 3–6	1.48	(1.05, 1.90)	
	0–3 vs. 4–6	1.31	(0.93, 1.69)	
	0–4 vs. 5–6	1.16	(0.77, 1.55)	
	0–5 vs. 6	1.03	(0.61, 1.45)	
ACAT	mRS at 90 d	OR =		0.0163
	0 vs. 1	1.12	(0.64, 1.97)	
	1 vs. 2	2.35	(1.25, 4.44)	
	2 v.s 3	0.7	(0.36, 1.38)	
	3 vs. 4	1.3	(0.73, 2.32)	
	4 vs. 5	0.79	(0.38, 1.66)	
	5 vs. 6	1.08	(0.52, 2.21)	
Logistic regression of sliding dichotomy	mRS at 90 d (0 if NIHSS is 1–7, 0–1 if 8–14 and 0–2 if >14)	OR = 1.61	(1.13, 2.28)	0.008
Linear regression of UW-mRS	UW-mRS at 90d	Diff. in means = 0.08	-	0.0175
Repeated measures GEE	mRS at 7–10, 90, 180 and 360 d (0–1 vs. 2–6)	OR = 1.89	(1.36, 2.63)	0.0002
Repeated measures GEE (with baseline)	Predicted mRS at baseline and mRS at 7–10, 90, 180 and 360 d (0–1 vs. 2–6)	OR = 1.78	(1.33, 2.38)	0.0001
MSMM (without baseline)	mRS at 7–10, 90, 180 and 360 d	Hazard Ratio =		0.0533
	0→1	0.72	(0.40, 1.30)	
	1→2	0.46	(0.23, 0.93)	
	2→3	3.04	(0.98, 9.41)	
	3→4	0.71	(0.34, 1.49)	
	4→5	0.9	(0.36, 2.23)	
	4→6	0.98	(0.50, 1.91)	
	5→6	1.69	(0.97, 2.95)	
	1→0	0.99	(0.60, 1.64)	
	2→1	1.03	(0.63, 1.70)	
	3→2	1.58	(0.64, 3.92)	
	4→3	0.99	(0.64, 1.53)	
	5→4	0.58	(0.32, 1.05)	
Piecewise MSMM (with baseline)	Predicted mRS at baseline and mRS 7–10, 90, 180 and 360 d	Hazard Ratio =		0.0018
	0→1	0.73	(0.43, 1.23)	
	1→2	0.51	(0.28, 0.90)	
	2→3	1.29	(0.66, 2.52)	
	3→4	0.67	(0.38, 1.17)	
	4→5	0.88	(0.53, 1.48)	
	4→6	0.96	(0.55, 1.66)	
	5→6	1.2	(0.75, 1.92)	
	1→0	1	(0.62, 1.62)	
	2→1	1.14	(0.71, 1.83)	
	3→2	0.99	(0.59, 1.68)	
	4→3	1.09	(0.72, 1.65)	
	5→4	0.8	(0.52, 1.23)	

**Table 3:** Summary of statistics obtained from each type of analysis of the mRS.

Method	Statistic(s)	Interpretation
Logistic regression	OR	The odds of good outcome in the treatment group versus placebo
Linear regression	Difference of means	Improvement of the average mRS score in patients that received treatment
Shift analysis	Probability value (no effect size or OR)	The treatment group shifted in a favorable direction toward a better mRS score versus placebo
POM	Summary odds ratio	The odds of a lower mRS the treatment group versus placebo
PPOM	ORs for six possible dichotomizations of mRS	Treatment has a significant benefit for certain definitions of good outcome
ACAT	ORs the six adjacent categories of response	The treatment group is more likely to have smaller mRS for certain adjacent mRS scores
Logistic regression of sliding dichotomy	OR	The odds of good outcome (defined by baseline severity) in the treatment group versus placebo
Linear regression of UW-mRS	Difference of mean utility scores	Improvement of the average utility score in patients that received treatment
Repeated measures GEE (dichotomized)	OR	The odds of good outcome over the 12-month period in the treatment group versus placebo
MSMM	Hazard ratios for each allowable transition	The hazard (instantaneous risk) of transitioning from one mRS state to another in the treatment group versus placebo

In the MSMM with baseline mRS, treatment with t-PA significantly reduced the transition intensity between mRS = 1 and mRS = 2 with a hazard ratio of 0.51 [95% CI: 0.28, 0.90]. None of the other hazard ratios were significantly different from one. This finding is consistent with the results of the ACAT model where the only significant OR is the one comparing mRS category 2 to mRS category 1. The conclusion drawn from the ACAT is that the most relevant impact of t-PA is to reduce the odds of observing a category 2 versus a category 1 at 90 days [6]. Although the results from the MSMM must be interpreted with caution, because they derive from secondary unplanned analysis, they might suggest a more refined conclusion- the most relevant impact of t-PA is to reduce the hazard of transitioning from mRS category 1 to mRS category 2. Therefore, the t-PA is more protective of worsening from category 1 rather than promoting improvement from category 2, which is a distinction that cannot be made from the ACAT results.

## Discussion

It is not realistic to choose one analytic method that is most appropriate for the mRS for all studies because the efficiency varies depending on the expected distribution of the treatment effect [26]. Here, efficiency refers to a test’s power to detect a difference when it truly exists, resulting in smaller sample sizes. In general, ordinal approaches are more efficient when treatment effects are distributed over the entire outcome range or when the distribution of treatment effect could not be pre-specified [26]. Therefore, it is important to know what the expected result of intervention is in the design and sample size calculation stage of a trial. In comparison, dichotomous approaches are more efficient than ordinal approaches when treatment effects cluster at single-state transitions and can be specified in advance [25].

In this paper, the dichotomized methods were found to be most statistically efficient with respect to power for the NINDS t-PA trial, and inclusion of predicted baseline mRS improved the ability to detect a treatment effect in the repeated measures analysis. The treatment effect clustered at the transition from mRS category 1 to mRS category 2. If limited information were available in the planning stages for this trial to confidently predict that the treatment effect would be clustered at that transition, it would have been worthwhile to consider an ordinal approach. Acute stroke trials are challenging to conduct as there are few centers that can recruit many patients in the early time window required for treatment [3]. Because of the low recruitment rate and cost associated with conducting acute stroke trials, inefficient statistical tests must be avoided to protect from being underpowered.

Of the approaches that do not rely on the strict dichotomy, the PPOM and MSMM with predicted baseline were the most efficient. The PPOM and MSMM were found to be more efficient than linear regression, responder analysis, shift analysis and the UW-mRS for analysis of the NINDS t-PA data. The PPOM is represented by ORs for the six possible dichotomizations of the mRS. The first three ORs are significantly different from one indicating that treatment has a significant benefit whether 0, 0–1 or 0–2 is defined as good outcome.

The MSMM without predicted baseline, while marginally significant, failed to detect a statistically significant treatment effect and is likely underpowered (p = 0.053). Including the estimated baseline mRS in the model allowed for detection of a statistically significant effect (p = 0.0018). This may be supportive of the use of the estimated baseline mRS since much progression and recovery occur early during the acute post stroke stage. Construction of MSMMs provides a more comprehensive view of the disease process and allow for exploration of how covariates affect the movement of the process. The obvious benefit to using the MSMM is the ability to handle progression and recovery simultaneously by estimating transition rates for both. Because of this, the MSMM allows for identification of where the treatment effect has the greater impact. Here the effect of treatment was greatest in reducing the hazard of transitioning from mRS category 1 to mRS category 2. A more clear understanding of the effect of treatment could also be beneficial in identifying characteristics of subjects that are more likely to benefit or experience harm from a therapy.

The MSMM results in a more comprehensive understanding of treatment effect; however it also increases the difficultly to determine the sample size to adequately power a study using this analysis. Without a summary statistic of effect size, the implementation of these models in the analysis of the primary outcome in trials requires quite a bit of foresight on the expected distribution of the effect of a therapy or treatment. However, once the distribution of expected treatment effect has been specified simulation-based power analysis for these models is straightforward. Another limitation of MSMMs is the increasingly computationally intensive nature as covariates and time-varying intensities are added to the models.

One notable feature of the MSMM for panel data is that observations do not have to be equally spaced. This is important for modeling acute stroke therapy trial data as the follow-up is often not scheduled in evenly spaced intervals. In the current example, the first observed mRS was at 7–10 days but the jitter for the other visits (90, 180 and 360 days) was ± 10 days. With the variability in measurement, the jitter is not equal. However, dropping the 7–10 day observation from the model did not change the results significantly, and the same conclusions can be drawn from models with or without this visit included.

Future directions of this research may include development of a software package to automate the baseline mRS prediction. The package could include more complex methods for estimation, potentially Bayesian PCA. Another feature of the package could be assistance with data manipulation required to use the *msm* package in R to fit the MSMMs (eg. wide to long format and incorporating exact time of death). In addition, a package could be developed to streamline the simulation-based power analyses including an examination of how many assessments over time are required for future trials.
